# Epigenetic dysregulation of steroidogenesis and neuroactive steroid deficiency in premature ovarian insufficiency: implications for neurodegenerative risk

**DOI:** 10.1186/s40364-025-00847-2

**Published:** 2025-11-13

**Authors:** Qian Wang, Junyan Sun, Lulu Wang, Xuefeng Lin, Liutong Wei, Qiuwan Zhang, Shuang Yuan, Dedong Xin, Dongmei Lai

**Affiliations:** 1 The International Peace Maternity and Child Health Hospital, School of Medicine, Shanghai Jiao Tong University, Shanghai, 200030 PR China; 2 Shanghai Key Laboratory of Embryo Original Diseases, Shanghai, 200030 PR China; 3 College of Life Sciences, Zhejiang Normal University, Jinhua, 321004 PR China

**Keywords:** Premature ovarian insufficiency, Dehydroepiandrosterone (DHEA), Pregnenolone, SOAT1, Neurodegenerative risk

## Abstract

Premature ovarian insufficiency (POI) is associated with an increased risk of neurodegenerative diseases, but the underlying mechanisms remain unclear. Here, we integrated DNA methylome profiling of peripheral blood leukocytes and circulating steroid hormone analysis to identify potential mechanism linking POI to neurogenerative risk. Methylome analysis revealed distinct epigenetic signatures in POI patients, including hypomethylation at the SOAT1 promoter, a gene critical for cholesterol homeostasis. Gene set enrichment analysis (GSEA) implicated suppressed steroid biosynthesis, supported by significantly reduced circulating levels of steroids, including androstenedione, dehydroepiandrosterone (DHEA), aldosterone, cortisol, and cortisone in POI patients. Notably, neuroprotective steroids DHEA and pregnenolone exhibited an age-dependent decline exclusively in the POI group. Our findings suggest that SOAT1-mediated cholesterol dysmetabolism leads to steroidogenesis suppression and depletion of neuroprotective steroids. Epigenetic dysregulation of SOAT1 and steroidogenic genes, coupled with depletion of DHEA and pregnenolone might contribute to the elevated neurodegenerative risk in POI.

**Supplementary Information:**

The online version contains supplementary material available at 10.1186/s40364-025-00847-2.

To the editor,

Premature ovarian insufficiency (POI), characterized by the loss of ovarian function before age 40, adversely affects fertility, bone and cardiovascular health, and neurological well-being [1]. POI is associated with an increased risk of dementia, supported by neuroimaging evidence of structural brain alterations [2]. However, the underlying mechanisms linking POI to neurodegeneration remain unclear. Through DNA profiling of peripheral blood leukocytes and subsequent analysis of circulating neuroactive hormones, we identified systemic molecular markers that may connect POI to neurodegenerative risk.

This study included 50 POI patients and 50 age-matched controls, divided into two cohorts. Methylome analysis was performed on Cohort 1 (20 POI patients, 20 controls). The flow chart of DNA methylation analysis was shown in Supplementary Figure F1. Steroid hormone quantification was conducted on cohort 2 (30 POI patients, 30 controls). The diagnostic criteria for POI were described in Supplementary Methods. As expected, POI Patients exhibited significantly elevated plasma follicle-stimulating hormone (FSH) and luteinizing hormone (LH) levels, alongside reduced ovarian reserve markers estradiol (E₂) and anti-Mullerian hormone (AMH) (Supplementary Table T1-T2).

Prior to principal component analysis (PCA) of the methylome data, we adjusted for slide effects while retaining age as a covariate, given known age-related methylation changes. PCA revealed PC2 (4.9% variance) and PC3 (3.8% variance) significantly correlated with case-control status (Group, Fig. 1B), enabling clear separation of POI patients from controls (Fig. 1C). PC1 has the largest variance (19% variance), primarily reflected leukocyte heterogeneity, showing a positive correlation with CD8 + memory T cell proportions (*r* = 0.78, *P* < 0.01), and a negative correlation with neutrophils proportions (*r* = −0.75, *P* < 0.01; Supplementary Figure F2), linking DNA methylation pattens to cellular composition.

**Fig. 1:**
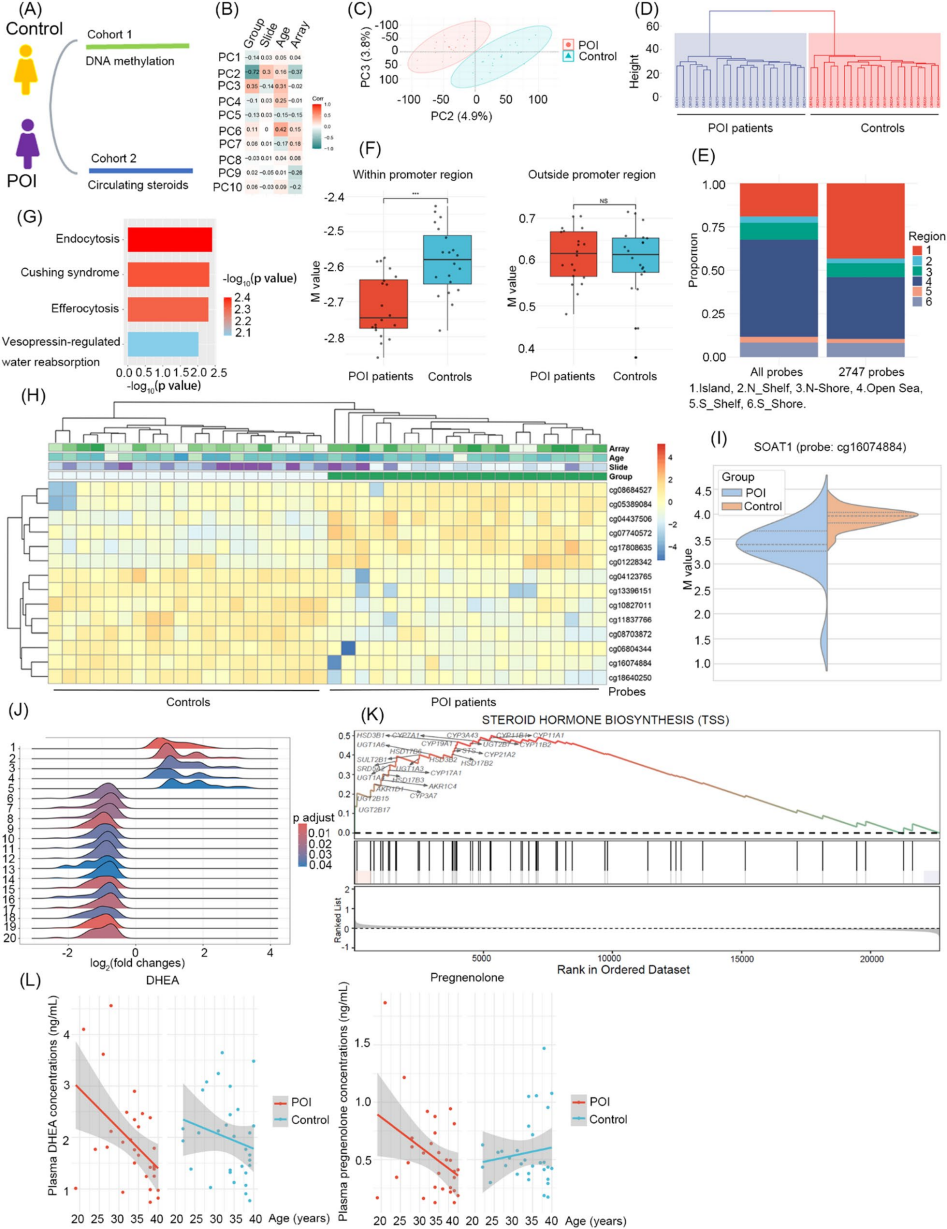
Epigenetic dysregulation of SOAT1 and steroidogenic genes, coupled with depletion of DHEA and pregnenolone in POI patients. (**A**) Schematic representation of two cohorts recruited in this study. (**B**) Correlation matrix of the PCs (principal component) with data features. Strong negative correlation between PC2 and Group (*r* = 0.72, *P* < 0.01). A weaker but statistically significant correlation between PC3 and Group (*r* = 0.35, *p* = 0.02). (**C**) Plot of the PC2 vs. PC3 showing complete group separation. (**D**) Hierarchical clustering of samples by 2747 probes with ≷ ± 4σ loadings in PC2 distinguishing POI patients from controls. (**E**) Distribution of PC2 probes across regions. The majority of PC2 probes were located in CpG islands (1,189 of 2,747; *P* < 0.01 by hypergeometric test). (**F**) Methylation pattern within or outside promoter region (TSS200, TSS1500, 1stExon, or 5’UTR). NS, not statistically significant; ***P < 0.001. (**G**) KEGG (Kyoto Encyclopedia of Genes and Genomes) enrichment analysis based on the genes corresponding to 2747 probes. Statistical differences were calculated between groups using Wilcoxon rank-sum test. (**H**) Heatmap based on 14 minimal set of probes. 14 probes were selected by Boruta algorithm and subsequent recursive feature elimination coupled with 5-fold cross-validation (AUC = 0.92, 95% CI: 0.88–0.96). Rows represent individual probes, color intensity reflects M value, columns represent samples, and corresponding Goup, Slide, Array and Age. (**I**) Violin plot showing methylation pattens of SOAT1 (probe: cg16074884) located at promotor region. The promoter of SOAT1 was hypomethylated in POI patients compared to controls. (**J**) Gene Set Enrichment Analysis (GSEA) of DNA methylation profiles. Ridge plot displaying significantly enriched KEGG pathways based on median methylation levels at transcription start sites (TSS). Pathways are ranked by their normalized enrichment scores (NES). (**K**) GSEA of the KEGG Steroid Hormone Biosynthesis pathway, based on promoter DNA methylation profiles in leukocytes. (**L**) Pearson’s correlation coefficient analysis was performed to evaluate the association between age and circulating concentrations of DHEA and pregnenolone in POI patients (*n* = 30) and age-matched controls (*n* = 30). DHEA POI group, *R* = −0.48, *P* = 0.0073; DHEA control group, *R* = −0.22, *P* = 0.25. Pregnenolone POI group, *R* = −0.39, *P* = 0.033; Pregnenolone control group, *R* = 0.13, *P* = 0.5

We identified 2,747 probes (|loading| > 4 standard deviations) in PC2 that distinguished POI patients from controls (Fig. 1D). Most of the probes (1,189 of 2,747) mapped to CpG islands (Fig. 1E), with promoter-associated region predominantly hypomethylated in POI group (Fig. 1F). Functional enrichment analysis implicated immune-metabolic dysregulation (Fig. 1G). Further refinement via the Boruta algorithm and random forest analysis selected 14 probes from 2747 probes as significant discriminators between POI patients and controls (Fig. 1H). Eleven of these probes mapped to known genes (Supplementary Table T3). Notably, SOAT1 (Sterol O-Acyltransferase 1, aka ACAT1), converting free cholesterol to cholesterol esters for storage in lipid droplets, was captured using probe cg16074884, with promoter hypomethylation in POI patients (Fig. 1I).

Since free cholesterol rather than cholesterol esters is the substrate for steroidogenesis [3], SOAT1 promoter hypomethylation may disrupt cholesterol homeostasis, impairing steroid biosynthesis. Supporting this, gene set enrichment analysis (GSEA) revealed promoter hypermethylation in steroid hormone biosynthesis pathway in POI patients (Fig. 1J-K), indicating steroid hormone biosynthesis is suppressed in POI patients.

We next quantified 18 circulating steroid hormones. Age-adjusted mean differences in hormone levels were summarized in Table 1. Statistically significant decreases were observed for androstenedione, dehydroepiandrosterone (DHEA), aldosterone, cortisol, and cortisone, alongside a modest increase in 18-hydroxycorticosterone. Notably, DHEA and pregnenolone concentrations in the POI group exhibited a significant age-dependent decline. In contrast, no significant correlation between age and these hormone levels was detected in the control group (Fig. 1L). DHEA and pregnenolone possess significant neuroprotective properties. DHEA combats oxidative stress, inflammation, apoptosis, and excitotoxicity while promoting neuronal survival and function. Pregnenolone serves as a precursor to other protective steroids, and modulates neurotransmitter systems. Both act as sigma-1 receptor agonist, collectively decreasing inflammation, reducing excitotoxicity, and attenuating oxidative stress [4–7]. The deficiency of DHEA and pregnenolone in POI patients might thus exacerbate neurodegenerative risk.

**Table 1:** Circulating steroid hormone concentrations in controls and patients with premature ovarian insufficiency (POI)

Steroid hormones	difference in age-adjusted means (POI vs. Control)	*P* value
Androstenedione	−0.46 (ng/mL)	**<** 0.001
Dehydroepiandrosterone (DHEA)	−0.09 (ng/mL)	< 0.05
Aldosterone	−0.15 (ng/mL)	< 0.05
Cortisol	−0.51 (ng/mL)	< 0.05
Cortisone	−2.95 (ng/mL)	< 0.05
18-hydroxycorticosterone	0.02 (ng/mL)	< 0.05
Androsterone	−0.20 (ng/mL)	NS
21-deoxycortisol	0.32 (ng/mL)	NS
17-hydroxyprogesterone	0.18 (ng/mL)	NS
Corticosterone	0.39 (ng/mL)	NS
Dehydroepiandrosterone sulfate (DHEAS)	0.05 (µg/mL)	NS
Estrone	0.18 (ng/mL)	NS
Testosterone	−0.03 (ng/mL)	NS
11-deoxycorticosterone	0.02 (ng/mL)	NS
Pregnenolone	−0.04 (ng/mL)	NS
Progesterone	0.45 (ng/mL)	NS
11-deoxycortisol	0.06 (ng/mL)	NS
Estriol	−0.35 (ng/mL)	NS

*NS* Not significant

While our findings highlight epigenetic and hormonal alterations that may predispose POI patients to neurodegeneration, this study did not include cohorts with diagnosed neurodegenerative diseases or track clinical outcomes. Future longitudinal studies are needed to validate whether SOAT1 hypomethylation and neurosteroid depletion predict neurodegenerative risk. The second limitation is that specific genes’ methylation in immune cells involved in steroid synthesis is still unclear. Future analyses of immune cells using single-cell transcriptomics or single-cell proteomics are expected to provide valuable insights into this issue.

In summary, Our findings implicate that epigenetic dysregulation of SOAT1 and steroidogenic genes, coupled with depletion of neuroprotective steroids DHEA and pregnenolone, as potential contributors to POI-associated neurodegenerative risk, offering targets for further investigation.

## Supplementary Information

Supplementary Material 1. Supplementary Methods.

Supplementary Material 2. Supplementary Figure F1.

Supplementary Material 3. Supplementary Figure F2.

Supplementary Material 4. Supplementary Table T1.

Supplementary Material 5. Supplementary Table T2.

Supplementary Material 6. Supplementary Table T3.

Supplementary Material 7. [8–12].

## Data Availability

The data involved in this study are available from the corresponding author upon reasonable request.

## Abbreviations

POI: premature ovarian insufficiency
FSH: follicle stimulating hormone
LH: luteinizing hormone
AMH: anti-Mullerian hormone
DHEA: dehydroepiandrosterone
SOAT1: Sterol O-Acyltransferase 1
