# Supplementary material for: Epigenetic dysregulation of steroidogenesis and neuroactive steroid deficiency in premature ovarian insufficiency: implications for neurodegenerative risk
Source: Biomark Res. 2025 Nov 13;13:147. doi: 10.1186/s40364-025-00847-2 (PMC12613854; doi:10.1186/s40364-025-00847-2)
Supplement: Supplementary file 1 — Supplementary Material 1. Supplementary Methods. [file 40364_2025_847_MOESM1_ESM.docx]

**Supplementary Methods**

## Participants

This study enrolled 50 patients with idiopathic POI and 50 controls from the International Peace Maternity and Child Health Hospital (IPMCH), Shanghai Jiao Tong University School of Medicine (July 2017–Aug 2021). Ethical approval for this study was granted by the Medical Ethics Committee of the IPMCH ((GKLW) 2018-43 and ((GKLW) 2022-62). All participants were made fully aware of the purpose of study and provided informed consents. All patients with POI were newly diagnosed who had not yet undergone hormone replacement therapy. The criteria for idiopathic POI included secondary amenorrhea for at least 4 months, and serum basal FSH > 25 IU/L (on two occasions separated by > 1 month) before age 40. Women with regular menstrual cycles and normal FSH level (< 10 IU/L) for regular examination were recruited as controls, and were matched for age and Body Mass Index (BMI), calculated based on height and weight measured by trained health professionals working at hospital. Exclusion criteria included women with a chromosomal abnormality, known gene mutations (such as fragile X messenger ribonucleoprotein 1 (FMR1) pre-mutation), history of ovarian or uterus surgery, radio or chemo-therapy, endometriosis or autoimmune disease, and history of cardiovascular, cerebrovascular, liver, kidney, and hematopoietic system or mental illness. Both patients with POI and controls who had been taking hormone therapy were also excluded.

## Peripheral blood plasma and leukocytes collection

Blood samples were obtained from control participants between days 2 and 4 of the menstrual cycle, whereas samples from POI participants were obtained at a random time point due to amenorrea. All samples were drawn from the median cubital vein. Whole blood samples were collected into commercially available EDTA-treated tubes. Plasma was separated from cellular components by centrifugation for 10 min at 1000g. The remaining cellular components were resuspened with red blood lysis buffer (Boster Biotech, China) for 20 min to lyse erythrocytes and centrifuging at 1000g. The cell pellet was washed twice with PBS buffer. The plasma and leukocytes were aliquoted and stored at -80℃ for downstream analyses.

## DNA methylation analysis

## M-value analysis

Genomic DNA was isolated from leukocytes and subjected to genome-wide methylation profiling using the Illumina Infinium HumanMethylationEPIC (850K) BeadChip (performed by OE Biotech, Shanghai, China). The flowchat of DNA methylation analysis was shown in Supplementary Figure F1. IDAT files were loaded into R (v4.2.0) (RRID:SCR_001905) using the minfi package [1]. Background adjustment and normalization was conducted with "preprocessIllumina" from the minfi package. Probes were filtered if 1) P-value ≥ 0.01; 2) being reported as cross-reactive; 3) single nucleotide polymorphisms at at CpG sites using the minfi function dropLociWithSnps. Methylation levels were quantified as M-values. M-value is defined as log_2_((meth + 100)/(unmeth + 100)), where meth and unmeth are the intensities of the methylated and unmethylated probes from the processed data, respectively. Probe-to-gene mappings were retrieved from the 'IlluminaHumanMethylationEPICanno.ilm10b4.hg19' Bioconductor packages for EPIC array data.

**Principal components** **analysis (PCA)**

PCA was performed on M-values using the prcomp function in R (centered but not scaled) (RRID:SCR_014676) to assess batch effects and identify major sources of variation in methylation profiles. Cluster analysis was performed with the R function dist (method = 'manhattan') followed by hclust (method = 'ward.D'). Results were visualized using ggplot2 (RRID:SCR_014601). The top ten principal components (PCs) were tested for correlations with 1) biological variables (case vs. control status (Group), Age); 2) technical variables (Slide, Array). Batch effect correction was performed using ComBat, , an empirical Bayes approach implemented in the R library sva.

**Leukocyte subtype profiling**

The cellular composition of blood leukocytes was inferred using the R package EpiDISH (v2.0) (RRID:SCR_018004) with the RPC (Robust Partial Correlation) method. A predefined reference matrix (cent12CT.m), comprising methylation profiles of 12 leukocyte subtypes for EPIC arrays, was employed to estimate subtype fractions.

**Functional enrichment analysis**

To account for potential redundancy arising from multiple probes mapping to the same gene, we aggregated probe-level data to the gene level by averaging methylation values across all probes associated with each gene. Gene symbols were then used for functional enrichment analysis via Metascape (RRID:SCR_016620) [2] to identify biologically relevant pathways.

**Selection of most discriminatiory probes**

The Boruta algorithm (RRID:SCR_016234) - a robust Random Forest-based wrapper method was applied to systematically identifie all statistically relevant probes. Further refinement was achieved through recursive feature elimination (RFE) with 5-fold cross-validation (implemented via replicate (5, rfcv)) to determine the most discriminatiory probes. The predictive utility of this refined probe set was validated through construction of a Random Forest classifier.

**Gene Set Enrichment Analysis (GSEA) of coordinated methylation changes**

Differential methylation was assessed using linear regression models (limma R package), adjusting for age as a covariate. A gene list, ranked by the moderated t-statistics from the limma lmfit output (reflecting the significance and direction of differential methylation), was generated. To ensure a single value per gene for GSEA (RRID:SCR_003199), when multiple probes mapped to the same gene, their t-statistics were aggregated by taking the median t-statistic. GSEA was subsequently performed on this ranked gene list, querying a collection of metabolic pathways, utilizing the clusterProfiler R package (RRID:SCR_016884) querying metabolic pathway gene sets from the Molecular Signatures Database (MSigDB v7.2) (RRID:SCR_016863) [3]. Enrichment results were visualized as dot plots using the enrichplot package (RRID:SCR_026996) [4].

## Steroid hormones analysis

Steroid hormones analysis was performed using liquid chromatography tandem mass spectrometry (LC–MS/MS) by Biozon Medical Laboratory Co. (Hangzhou, China) with an ACQUITY® UPLC I-Class IVD / Xevo® TQ-S IVD system (Waters Corporation，USA). 10 μL of the dissolved samples were injected and separated on a ACQUITY UPLC HSS T3 (1.8 μm 2.1 × 50 mm) column (Waters Corporation，USA) at a temperature of 45℃. Mobile phase A consisted of methanol/water (5/95; v/v), mobile phase B was 100% methanol, both containing 0.1% formic acid and 2 mmol/L ammonium acetate. MS raw files were analyzed using MassLynx software (version V4.1 SCN950，Waters Corporation，USA) (RRID:SCR_014271). The precision at the steroid hormone levels was set at < 10%, and the recovery was set at 85%-115%.

## Statistical analysis

Probes were filtered via minfi::dropLociWithSnps with p-value < 0.01. Background correction and normalization were performed via minfi::preprocessIllumina. PCA was conducted on M-values (prcomp; centered, not scaled). Identification of technical (Slide/Array) vs. biological (Group/Age) variance was assessed using Kendall’s τ correlation coefficient (psych::corr.test). ComBat (sva package) (RRID:SCR_010974) was applied to remove slide effects while retaining age as a covariate. Leukocyte fractions were estimated via EpiDISH (RPC method; reference: cent12CT.m). CpG island enrichment was tested via hypergeometric distribution (phyper). Hierarchical clustering (Manhattan distance, Ward’s D method) (RRID:SCR_014673) was used to validate group separation. Boruta Algorithm and 5-fold cross-validation (rfcv) refinement was applied to identified relevant probes. For Gene Set Enrichment Analysis (GSEA), significance thresholds were set at |NES| > 1, p < 0.05, and FDR ≤ 0.25. For mean comparisons, the Wilcoxon rank-sum test was used and the Bonferroni correction was applied for multiple comparisons. Steroid concentration analysis was conducted using  R package limma with age included as a covariate. Pearson correlation coefficient was applied to assess the correlations between DHEA or pregnenolone with age.
