# Supplementary figures and images for: Epigenetic dysregulation of steroidogenesis and neuroactive steroid deficiency in premature ovarian insufficiency: implications for neurodegenerative risk
Source: Biomark Res. 2025 Nov 13;13:147. doi: 10.1186/s40364-025-00847-2 (PMC12613854; doi:10.1186/s40364-025-00847-2)

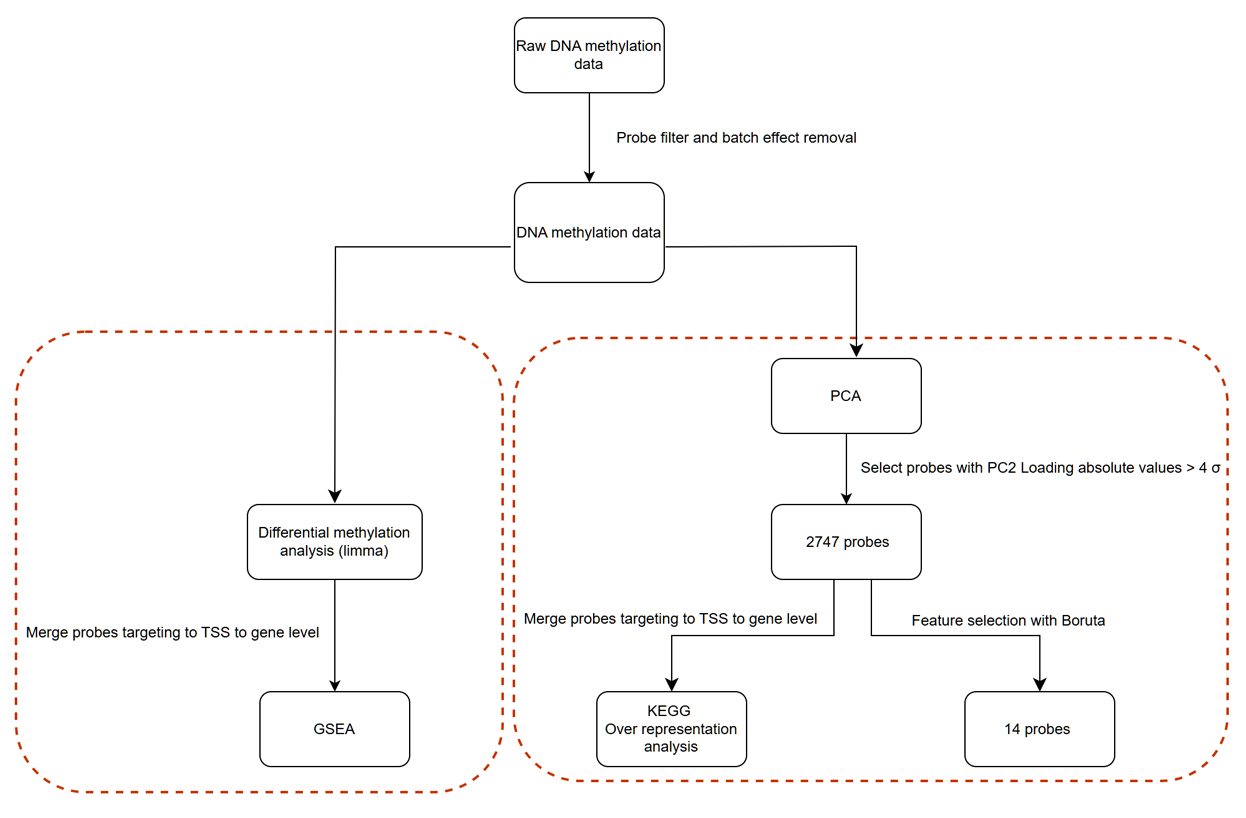

Supplement: Supplementary file 2 — Supplementary Material 2. Supplementary Figure F1. [file 40364_2025_847_MOESM2_ESM.tif]

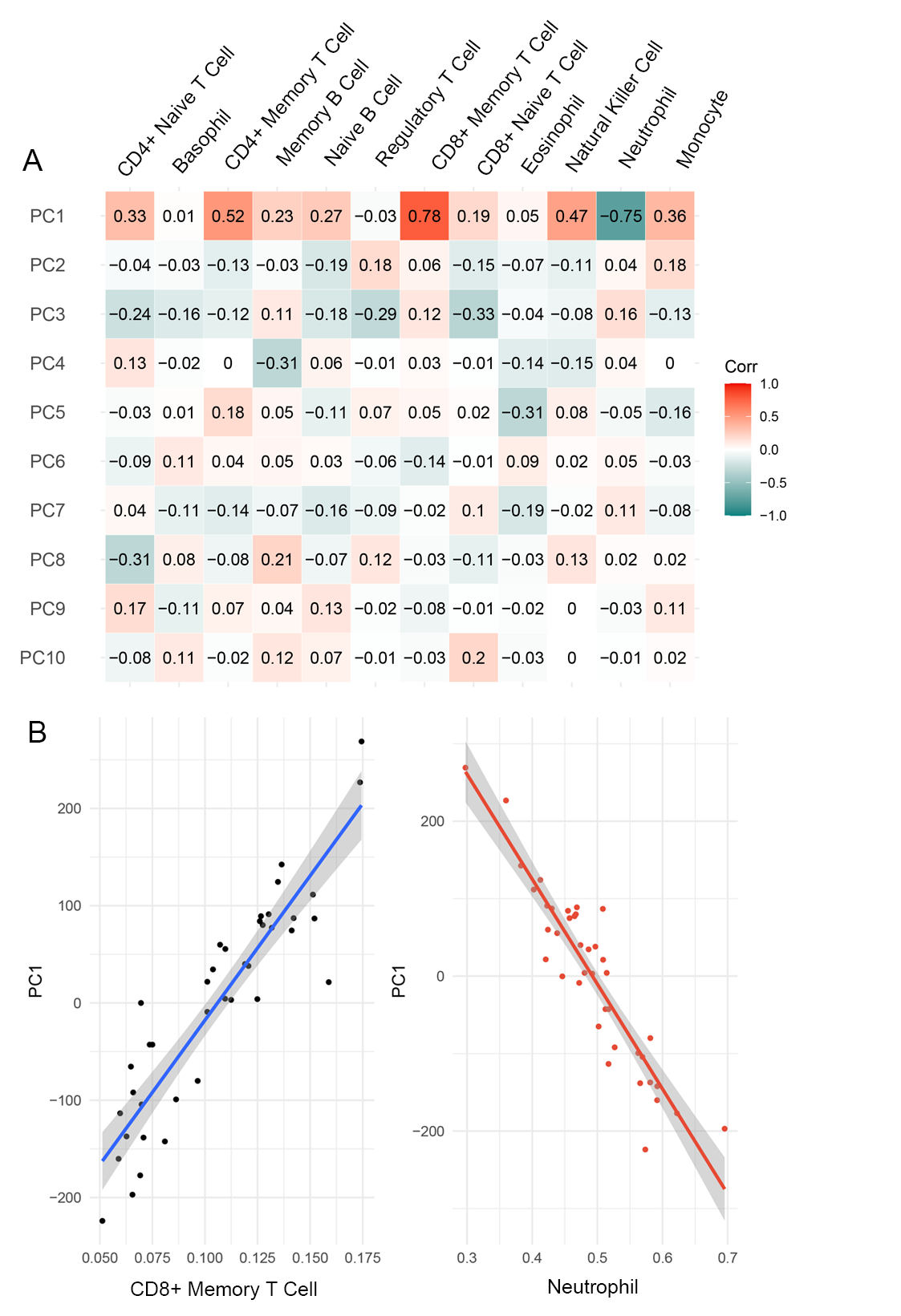

Supplement: Supplementary file 3 — Supplementary Material 3. Supplementary Figure F2. [file 40364_2025_847_MOESM3_ESM.tif]
