# Supplementary material for: Epigenetic dysregulation of steroidogenesis and neuroactive steroid deficiency in premature ovarian insufficiency: implications for neurodegenerative risk
Source: Biomark Res. 2025 Nov 13;13:147. doi: 10.1186/s40364-025-00847-2 (PMC12613854; doi:10.1186/s40364-025-00847-2)
Supplement: Supplementary file 4 — Supplementary Material 4. Supplementary Table T1. [file 40364_2025_847_MOESM4_ESM.docx]

**Supplementary Table T1. Clinical characteristics of the participants undergoing DNA methylation profiling in peripheral blood leukocytes**

| Information | Control (n = 20) | POI (n = 20) | *P* value |
| --- | --- | --- | --- |
| Age (years) | 34.2 ± 0.2 | 34.3 ± 0.2 | NS |
| BMI (kg/m^2^) | 20.4 ± 0.1 | 20.6 ± 0.1 | NS |
| FSH (IU/L) | 6.64 ± 0.06 | 94.23 ± 1.63 | ＜ 0.001 |
| LH (IU/L) | 3.79 ± 0.07 | 42.09 ± 1.04 | ＜ 0.001 |
| E2 (pmol/L) | 276.9 ± 8.6 | 76.2 ± 3.1 | ＜ 0.001 |
| AMH (ng/mL) | 1.938 ± 0.024 | 0.299 ± 0.016 | ＜ 0.001 |

Abbreviations: BMI, body mass index; POI, premature ovarian insufficiency; FSH, follicle stimulating hormone; LH, luteinizing hormone; AMH, anti-Mullerian hormone. The results are represent as mean ± SEM. NS, not statistically significant.
