# Supplementary material for: Epigenetic dysregulation of steroidogenesis and neuroactive steroid deficiency in premature ovarian insufficiency: implications for neurodegenerative risk
Source: Biomark Res. 2025 Nov 13;13:147. doi: 10.1186/s40364-025-00847-2 (PMC12613854; doi:10.1186/s40364-025-00847-2)
Supplement: Supplementary file 5 — Supplementary Material 5. Supplementary Table T2. [file 40364_2025_847_MOESM5_ESM.docx]

**Supplementary Table T2. Clinical characteristics of the participants undergoing LC-MS/MS steroid hormones analysis**

| Information | Control (n = 30) | POI (n = 30) | p value |
| --- | --- | --- | --- |
| Age (years) | 33.6 ± 0.2 | 33.1 ± 0.2 | NS |
| BMI (kg/m^2^) | 20.9 ± 0.1 | 21.4 ± 0.1 | NS |
| FSH (IU/L) | 7.04 ± 0.04 | 75.18 ± 1.07 | ＜ 0.001 |
| LH (IU/L) | 5.63 ± 0.07 | 34.89 ± 0.55 | ＜ 0.001 |
| E2 (pmol/L) | 273.5 ± 9.6 | 123.1 ± 3.5 | ＜ 0.05 |
| AMH (ng/mL) | 3.938 ± 0.101 | 0.169 ± 0.003 | ＜ 0.01 |

Abbreviations: BMI, body mass index; POI, premature ovarian insufficiency; FSH, follicle stimulating hormone; LH, luteinizing hormone; AMH, anti-Mullerian hormone. The results are represent as mean ± SEM. NS, not statistically significant.
