# Supplementary material for: Epigenetic dysregulation of steroidogenesis and neuroactive steroid deficiency in premature ovarian insufficiency: implications for neurodegenerative risk
Source: Biomark Res. 2025 Nov 13;13:147. doi: 10.1186/s40364-025-00847-2 (PMC12613854; doi:10.1186/s40364-025-00847-2)
Supplement: Supplementary file 6 — Supplementary Material 6. Supplementary Table T3. [file 40364_2025_847_MOESM6_ESM.docx]

**Supplementary Table T3. Differentially methylated probes and corresponding genes associated with premature ovarian insufficiency**

| Probe | Chromosome | Relation_to_  island | Gene symbol | Gene group | Source sequence |
| --- | --- | --- | --- | --- | --- |
| cg17808635 | Chr1 | Island | LMNA | Body | TTTTTTTTTCTTCCTTCTCTGGCCTCTATCTACCTAGTCCTGAAATGACG |
| cg11837766 | Chr1 | Island | ROR1 | TSS200 | CGGCCCAGGCTGGCGACCCAGCGCCCTCTGCTGCAGCCTCTCAACGCCCG |
| cg08703872 | Chr1 | Island | C2CD4D | 5’UTR | CGCCACCACGCGCTCGCCTCACCCCTGCGCGGAGCCACCAAGAGCACCGT |
| cg16074884 | Chr1 | OpenSea | SOAT1 | 5'UTR | TATAGCCCCAGCATCCAGCGGGGGACCTCAGCAATTATTGGTAGGTTACG |
| cg04123765 | Chr1 | OpenSea | OR2L13 | 5'UTR | TGGAAAATTACAATCAAACATCAACTGCTTTCATCTTGTTGGGATTGTCG |
| cg04437506 | Chr3 | Island | HDAC11 | TSS1500 | GCGGAGGCGGGACTTCGGGCTTGATGGGCGTTGGGGGTGGCCTTCCTGCG |
| cg01228342 | Chr3 | OpenSea | EIF4E3 | 5'UTR | CCATCTAGTGGCCATTGCCTTCCTTTTTCTTTTGCTGTTAAAGGAAAACG |
| cg06804344 | Chr3 | OpenSea | GP9 | TSS1500 | GAAGGCTGACGGAGGCTTTAAACGCACTTCGTTGACATTCAGGACCCTCG |
| cg08684527 | Chr3 | OpenSea | EPHB1 | Body | ACCTTGCTCCCTGTAGCATCTGTCTAAGTGTCAATGCTGTCATGTCAACG |
| cg05389084 | Chr3 | N_Shore | VEPH1 | TSS1500 | CGTGAGCCACCTCACCCGGCCTATGACAAACAACACAGTACAACTGGGTA |
| cg10827011 | Chr4 | Island | IDUA | TSS200 | CGGGCGAAGCGTTCTTCTGAGCGCTTTCCGAGTCATCGGTCCTCAGAGCA |
