# Supplementary material for: Epigenetic dysregulation of steroidogenesis and neuroactive steroid deficiency in premature ovarian insufficiency: implications for neurodegenerative risk
Source: Biomark Res. 2025 Nov 13;13:147. doi: 10.1186/s40364-025-00847-2 (PMC12613854; doi:10.1186/s40364-025-00847-2)
Supplement: Supplementary file 7 — Supplementary Material 7. [8–12]. [file 40364_2025_847_MOESM7_ESM.docx]

**Extended Discussion**

In this study, we reveal multi-level disruption of steroidogenesis in POI patients. First, hypomethylation at the promoter regions of SOAT1, a key regulator of cholesterol homeostasis, was identified. Second, Promoter hypermethylation in the steroid hormone biosynthesis pathway implies transcriptional silencing of key enzymes, such as CYP11A1, CYP17A1, HSD3B2 etc. Third, the epigenetic dysregulation is accompanied by consistent reductions in androgens (androstenedione, DHEA), mineralocorticoids (aldosterone), and glucocorticoids (cortisol, cortisone), indicating broad impairment across adrenal and gonadal steroidogenic axes.

POI is associated with an increased risk of cognitive impairment and dementia. Epigenetic dysregulation, such as non-coding RNA regulation, DNA methylation, and histone modifications, plays a critical role in premature follicular depletion, granulosa cell dysfunction, and ovarian aging. These processes contribute to POI [1]. Although analyses of peripheral blood DNA in POI patients demonstrate differential methylation patterns enriched in pathways critical for follicle development, steroid hormone biosynthesis, and immune regulation, no study has explored the potential link between hormone biosynthesis and neurodegenerative risk. Circulating 17β-estradiol, progesterone and other steroid hormones produced by endocrine glands can cross the blood-brain barrier to reach the brain. They are termed neuroactive steroids. Neuroactive steroids can also be synthesized in the brain de novo [3]. Neuroactive steroids have multiple neuroprotective effects. They can modulate neuroinflammation, promote myelin growth, and support neuronal adaptation. Neurodegenerative diseases like Alzheimer disease (AD), Multiple Sclerosis (MS), Parkinson’s disease (PD) and traumatic brain injury (TBI) are characterized by inflammatory activation of microglia and astrocytes [6, 7]. Free cholesterol serves as the initial substrate for steroid synthesis. Under the action of CYP11A1, free cholesterol is catalyzed to produce pregnenolone, the precursor to all other steroids [3]. SOAT1 catalyzes the conversion of free cholesterol into cholesteryl esters. Hypomethylation of SOAT1 in POI patients might impair steroid synthesis through interference of cholesterol homeostasis. Dysregulation of cerebral cholesterol biosynthesis and catabolism has been implicated in AD pathogenesis. Blocking SOAT1 in AD mouse models reduces β-Amyloid production, amyloid plaque burden, and cognitive deficits [8]. Disrupted cholesterol homeostasis and deficiency in neurosteroids may represent shared features between POI and neurodegenerative diseases. Our study integrates SOAT1 hypomethylation with steroid hormone deficiencies to propose a novel mechanistic link to neurodegenerative risk.

The brain is a target for ovarian steroid hormones such as 17β-estradiol, progesterone and follicle-stimulating hormone (FSH). Estradiol and progestone, particularly estradiol, support cognitive function through maintaining metabolic homeostasis in the brain, where it regulates glucose metabolism, glycolysis, oxidative phosphorylation and ATP generation in neurons and has neuroprotective effects by enhancing synaptic plasticity, reducing β-Amyloid, and decreasing oxidative stress and inflammation in the brain [1]. While FSH induces neuronal inflammation and apoptosis, leading to recognition memory loss [9]. Women with POI experience menopause 10-30 years earlier than average, resulting in prolonged estrogen deficiency and elevation of FSH. In our study, women with POI had substantially lower estradiol and higher FSH levels. For estradiol (POI women vs. controls), Cohort 1: 76.2 ± 3.1 pmol/L vs. 276.9 ± 8.6 pmol/L (*P* < 0.001); Cohort 2: 123.1 ± 3.5 pmol/L vs. 273.5 ± 9.6 pmol/L (*P* < 0.05). For FSH (POI women vs. controls), Cohort 1: 94.23 ± 1.63 IU/L vs. 6.64 ± 0.06 IU/L (*P* < 0.001). Cohort 2: 75.18 ± 1.07 IU/L vs. 7.04 ± 0.04 IU/L (*P* < 0.001) (Supplementary Table T1, T2).

To our knowledge, among the steroids we detected as altered in the plasma of POI patients, androstenedione has been reported to be significantly reduced in women with POI, with a standardized mean difference (SMD) of -1.09 and 95% CI [-1.71, -0.48] [10], which is consistent with our findings. For other steroids examined, no previous reports comparing their levels in POI patients versus healthy controls were retrieved. Current research on the regulation of ovarian function by steroid synthesis primarily relies on animal models and in vitro follicle culture techniques. In our analysis of plasma steroid concentrations, we included age as a covariate, allowing a more accurate reflection of the differences in steroid levels between POI patients and healthy controls. Notably, in POI patients, the concentrations of pregnenolone and DHEA showed a significant negative correlation with age, whereas no such association was observed in healthy controls. These findings represent an update to the existing knowledge.

The current study was performed on peripheral blood leukocytes. Immune cells, such as macrophages and T lymphocytes are capable of synthesizing steroids, as reviewed in [11]. Our results indicate that in POI patients, CD8+ memory T lymphocytes show positive correlation with the principal component 1 (PC1) of the methylome (Supplementary Figure F2). However, it remains uncertain whether this cell type actively synthesizes steroids in POI patients.

The primary organs responsible for steroid synthesis in women include the adrenal glands, ovaries, placenta (if pregnant), and the nervous system [11]. Although SOAT1 can influence steroid synthesis by regulating cholesterol homeostasis, each step of steroidogenesis is modulated by various other genes and metabolites. Therefore, the steroid levels detected in plasma reflect the coordinated effects of numerous genes and metabolites throughout the body. It is possible that steroids showing no significant changes in plasma may still undergo alterations within specific organs or tissues.

Considering their potential use as biomarkers, we believe that DHEA and pregnenolone may be more informative than other hormones. First, the initial step of steroidogenesis involves CYP11A1 catalyzing the conversion of free cholesterol into pregnenolone, which serves as the precursor for all other steroids [3]. Second, DHEA is clinically used to improve outcomes in women with poor ovarian response, benefiting patients undergoing assisted reproductive technology. Although a meta-analysis involving 1,533 women seeking fertility treatment and 1,469 controls showed that DHEA likely results in little to no difference in live birth/ongoing pregnancy rates or clinical pregnancy rates, certain individuals did experience benefits—achieving successful conception and childbirth after oral DHEA supplementation [12].

There are two limitations of this study: First, methylome analyses and hormonal assays were conducted in two independent cohorts. And it is still unclear which type of immune cells and which specific genes' methylation in immune cells influences steroid synthesis. Detection of plasma steroids, neurostructural changes, and single-cell transcriptomics or single-cell proteomics of immune cells should be performed together in the future. Second, the current study design does not include patients with diagnosed neurodegenerative conditions, and no prospective monitoring of neurodegenerative outcomes was performed. Collaborating with neurologists to identify eligible patients with a history of POI for further research and continue longitudinal follow-up of our existing POI cohort to monitor changes in their cognitive function are warranted.
